# Investigation of red blood cell mechanical properties using AFM indentation and coarse-grained particle method

**DOI:** 10.1186/s12938-017-0429-5

**Published:** 2017-12-19

**Authors:** Sarah Barns, Marie Anne Balanant, Emilie Sauret, Robert Flower, Suvash Saha, YuanTong Gu

**Affiliations:** 1 Science and Engineering Faculty, Queensland University of Technology (QUT), Brisbane, 4000 Australia; 2 Research and Development, Australian Red Cross Blood Service, Brisbane, 4059 Australia; 3 Faculty of Health, Queensland University of Technology, Brisbane, 4000 Australia

**Keywords:** Red blood cell, Atomic force microscopy (AFM), Indentation, Numerical model, Coarse-grained particle method

## Abstract

**Background:**

Red blood cells (RBCs) deform significantly and repeatedly when passing through narrow capillaries and delivering dioxygen throughout the body. Deformability of RBCs is a key characteristic, largely governed by the mechanical properties of the cell membrane. This study investigated RBC mechanical properties using atomic force microscopy (AFM) with the aim to develop a coarse-grained particle method model to study for the first time RBC indentation in both 2D and 3D. This new model has the potential to be applied to further investigate the local deformability of RBCs, with accurate control over adhesion, probe geometry and position of applied force.

**Results:**

The model considers the linear stretch capacity of the cytoskeleton, bending resistance and areal incompressibility of the bilayer, and volumetric incompressibility of the internal fluid. The model’s performance was validated against force–deformation experiments performed on RBCs under spherical AFM indentation. The model was then used to investigate the mechanisms which absorbed energy through the indentation stroke, and the impact of varying stiffness coefficients on the measured deformability. This study found the membrane’s bending stiffness was most influential in controlling RBC physical behaviour for indentations of up to 200 nm.

**Conclusions:**

As the bilayer provides bending resistance, this infers that structural changes within the bilayer are responsible for the deformability changes experienced by deteriorating RBCs. The numerical model presented here established a foundation for future investigations into changes within the membrane that cause differences in stiffness between healthy and deteriorating RBCs, which have already been measured experimentally with AFM.

**Electronic supplementary material:**

The online version of this article (10.1186/s12938-017-0429-5) contains supplementary material, which is available to authorized users.

## Background

The red blood cell (RBC) is composed of a membrane surrounding haemoglobin-rich fluid, to which dioxygen binds when the cell traverses the lungs. RBCs are responsible for distributing this dioxygen throughout the body, as well as removing waste products [1]. This requires RBCs to repeatedly pass through narrow blood vessels which can be less than half their own diameter, making deformability a key characteristic for RBCs to efficiently deliver dioxygen to bodily tissues [2].

The cell membrane is critical to the deformability of RBCs [3]. It is a composite of two main components—an outer lipid bilayer composed of phospholipids and cholesterol with embedded proteins, and a spectrin-based cytoskeleton tethered beneath. The bilayer and cytoskeleton are tightly bound through interactions between transmembrane proteins within the bilayer and proteins of the cytoskeleton [4]. From a mechanical perspective, the bilayer provides resistance to bending and thus restrains membrane curvature. The bilayer also resists changes in its surface area. The cytoskeleton facilitates stretch deformation through the folding and unfolding of spectrin proteins, which are long molecules that bind with actin at their ends to form a triangulated network beneath the bilayer [5]. RBC deformability is thus largely governed by the extent to which the membrane resists bending, stretch and areal changes, as well as the incompressibility of the cytoplasm.

In the past, the study of RBC deformability has largely focused on the experimental methods of micropipette aspiration, optical tweezer stretching and flow visualisation [6]. Another more recent technique for measuring physical characteristics of biological samples is atomic force microscopy (AFM), which involves a cantilevered probe applying a force onto a sample while displacement is measured. A major advantage of using AFM over other experimental methods is that force and deformation can be measured at different locations over the cell membrane with high accuracy: AFM provides control over the indentation point, meaning that force acting on the membrane can be quantified locally. AFM can be performed while cells are submerged in liquid, meaning experimental conditions can replicate aspects of the physiological environment and limit sample treatment [7].

A significant aspect of applying AFM to RBCs is managing the elasticity of the membrane during imaging and indentation, while protecting its natural organisation [8, 9]. Probe shape is an important consideration, with most AFM studies of RBCs using conical and pyramidal tips [8–13]. These sharp tips can push the membrane beyond physiological limits leading to penetration and rupture. To overcome these risks, spherical probes have been considered [14, 15]. Another challenge for AFM is attaching the cell to the substrate such that it is immobilised for imaging and indentation, while preserving the membrane mechanical properties. Poly-lysine is a chemical typically used for this purpose which causes bonding between the substrate and negative charges of the membrane surface proteins, however it can also cause membrane tension [16, 17]. To balance adhesion strength against preservation of the membrane’s natural state, the adhesion protocol needs to be carefully considered.

Hertz-based models have been widely used for analysing experimental RBC force-indentation data in order to estimate the stiffness of the membrane [8–13, 15]. This method of analysis has gained popularity in recent times, likely due to its simplicity which enables a standard equation to be routinely fitted to the experimental curves to extract an effective Young’s modulus of the RBC membrane. However, the trade-off of this analysis is that there are significant limitations in reasoning the assumptions of solid mechanics contact for biological samples [18]—Hertz-based equations were developed specifically for solid-to-solid contact of elastic, isotropic materials where the size of the contact region was negligible compared to the bodies themselves. Therefore it should be emphasised that the effective Young’s modulus is only a qualitative estimate of the membrane’s stiffness [18]. Nonetheless, previous studies have shown that Hertz-based empirical equations show close agreement for the experimental force–deformation trend at small indentations of biological samples [8–13, 15, 19, 20]. Furthermore, force–deformation results from AFM are sensitive enough to detect a difference in the effective Young’s modulus between normal RBCs and those from patients suffering from diabetes [10, 13], sickle cell disease [9] and cigarette smoking [10]. This has led to cell indentation being proposed as a possible diagnostic tool as mechanical properties of neoplastic cells have been shown to differ significantly from healthy cells [11, 15, 21]. Therefore Hertz-based equations can be considered in this study for the purpose of validating experimental results and as an empirical equation which describes the experimental force–deformation trend.

To investigate and understand the more fundamental mechanics of RBC deformability, it is desirable to develop numerical models. This allows investigation of the mechanical aspects that define RBC behaviour at a much smaller scale than is possible with experimentation, which becomes challenging and costly [22]. Studying elasticity of the RBC membrane at this level can provide insight on the state of the membrane and how structural changes and defects impact on physical characteristics of the cells [23]. Structural transformations within the membrane are known to occur naturally as RBCs age, also as a result of underlying conditions such as malaria, sickle cell trait and hereditary diseases [10, 13], and even during the storage of RBCs before transfusion [24]. Thus, better understanding of membrane changes may aid the development of measures which preserve RBC deformability. Using a numerical model can help isolate the effect of particular conditions on the different membrane components.

The most basic models previously applied for RBC indentation are the Hertz-based ones discussed above [9, 10, 13], which have restricted inputs (such as a geometric property of the probe and cell) and a singular output of effective Young’s modulus. To date, there is one model that simulates the overall shape of the probe and cell during indentation in the report of Sen et al. [18]. This is an analytical membrane model focused on membrane tension, which approximates the cell as a partial sphere with constant volume. Critically, it does not consider the bending resistance of the membrane which is cited as a major limitation of the study, as bending is known to contribute significantly to RBC properties. The model is also limited in its application, as it is only relevant for indentation and only for RBCs which have formed perfectly symmetrical dome shapes. It is therefore necessary to evolve beyond analytical frameworks to develop an advanced numerical model capable of simulating RBC behaviour during indentation, with versatility for investigating how the cell acts in extended scenarios.

Several numerical techniques have been applied in the past to model RBCs at rest as well as during flow, stretching and micropipette aspiration. These can be broadly categorised as finite element methods (FEM) [25–28] or particle-based methods, encompassing coarse-grained molecular dynamics (CGMD) [29, 30], dissipative particle dynamics (DPD) [31, 32] and the coarse-grained particle method (CGPM) (refer to Table 2 for comprehensive list of CGPM studies). Particle-based methods involve coarse-graining the membrane into interconnected regions. For CGMD, the elements are on a very small scale indicating an enormous number are required to discretise the system. This is likely why CGMD models have only simulated a small region of the RBC membrane. In contrast, the lower particle resolution of DPD and CGPM models has allowed efficient prediction of RBC deformability at the full cell scale. The key difference between DPD and CGPM is that a fluid phase is innately incorporated in DPD, while the CGPM can be coupled with smoothed particle hydrodynamics (e.g. [33–35]) or the immersed boundary method (e.g. [22, 36]) to simulate the fluid phase. Modelling fluids is much more difficult for FEM models which require high degrees of mesh refinement and more complex solid–fluid coupling which adds significant computational cost [27, 37]. Finally, particle-based methods have significant potential for investigating the effect of heterogeneity and defects within the membrane. This is because it is easy to introduce variance in regional properties or even between specific membrane particles [29], but is not possible with FEM. This indicates there is significant potential for particle-based methods to link the state of the membrane under different conditions to observed behaviour, and to thus develop deeper understanding for the role and importance of individual membrane components.

This study aims to develop foundation 2D and 3D numerical models for RBC indentation, validated for resting shape, adhered shape and the force–deformation response. The models will be applied to understanding how membrane stiffness properties impact on the observed behaviour. Given the numerical models represent a substantial improvement on the analytical model of Sen et al. [18] (by considering bending and affording control over adhesion, probe geometry and applied force position), it is expected that they can be further developed in the future to investigate more complex changes within the membrane that cause differences in stiffness between healthy and deteriorating RBCs, which have already been measured experimentally [9–11, 13, 15].

## Modelling methodology

### CGPM overview

From the literature review presented in the introduction, the advantages of the coarse-grained particle method (CGPM) make it the most suitable technique for the present developments. The method was first applied to RBCs by Tsubota et al. [38], and it has since been implemented in a number of subsequent studies (refer to Table 2 for comprehensive list).

The CGPM relies on discretisation of the membrane into particles which are interconnected by a network capable of storing energy. This network is developed to model the mechanical behaviour of cellular components—stretch resistance of the cytoskeleton, areal incompressibility of the bilayer, bending resistance of the bilayer, and volumetric incompressibility of the internal fluid. A stiffness coefficient is associated with each of these energy storing mechanisms. When total energy in the network is minimised, the preferred RBC resting shape is predicted.

Quantification of the stiffness coefficients is an absolutely critical aspect of the modelling, as they dictate accuracy of predictions [39]. However, there is significant variation in the coefficients used in existing CGPM models of RBCs (discussed in detail in “Comparison to stiffness coefficients of previous studies” section) and there is little evidence to justify their basis. This is because most CGPM models are only validated with qualitative comparison of the shapes RBCs exhibit at rest and during general flow conditions (e.g. [33, 40, 41]). The only exception is in the report of Shi et al. [42] who also simulated stretching. In the present study, stiffness coefficients for each energy storage mechanism are established for indentation in both 2D and 3D.

### Model initialisation and energy storage mechanisms

**Fig. 1:**
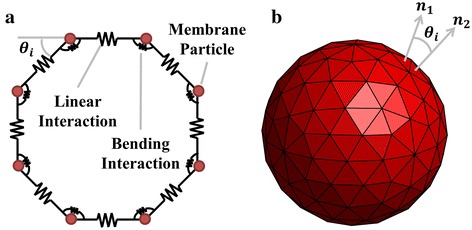
Model setup schematics **a** 2D for *N* = 8, **b** 3D for *N* = 122 where each vertex represents the position of a membrane particle, edges show linear interactions between adjoining particles and bending interactions are present between adjoining triangular surfaces

To initiate the computational model, *N* particles were evenly distributed around a 3 µm radius circle (2D) [38] and 3.3 µm radius sphere (3D) [43] as shown in Fig. 1. For the 3D case, this was done with the aid of a spherical surface mesh created with Comsol Multiphysics 4.4, composed of *N* vertices, *N*_*e*_ edges and *N*_*t*_ triangles. Each vertex represented the location of a membrane particle, each edge represented a linear interaction between adjoining particles, and bending interactions were present between adjoining triangular surfaces. On average, six triangles formed around each vertex, aligning with the cytoskeleton’s junctional complex structure [5].

The minimum energy RBC shape was obtained by moving particles over time with the principle of virtual work and Newton’s Second Law,

1 $$F_{i} = - {\frac{\partial E}{\partial s_{i} }} = ma_{i}$$

where *F*_*i*_ is the force on particle *i*, *E* is the total energy stored in the membrane expressed as a function of particle positions, and *s*_*i*_ the position of particle *i*. A mass, *m*, was used to convert force into an acceleration of each particle, *a*_*i*_. A time step was applied to calculate how far each particle moved over progressive iterations. A small amount of damping was applied to the particle velocities to gradually approach the steady-state shape. The time step, damping and mass do not impact on the steady-state shape, however they do contribute to the dynamics of how quickly the steady-state is achieved.

The equations for calculating stored energy are presented in Table 1. Total energy, *E*, is the sum of energy stored through stretch resistance of the cytoskeleton, surface area incompressibility of the bilayer, bending resistance of the bilayer, and volumetric incompressibility of the internal fluid. It can be seen that the 2D model only has three components for total energy (Eq. 2) compared to four for 3D (Eq. 3). This is because in 2D, areal incompressibility of the bilayer is effectively combined with spectrin’s stretch resistance to oppose the relative movement of adjacent particles.

**Table 1:** Energy equations used in the 2D and 3D models

Energy term	2D	3D
Total energy	$E = E_{l} + E_{b} + E_{a} \quad (2)$	$E = E_{L} + E_{A} + E_{B} + E_{V} \quad (3)$
Stretch resistance	$E_{l} = \mathop \sum \limits_{i = 1}^{N} \frac{{k_{l} }}{2}(l_{i} - l_{0,i} )^{2} \quad (4)$	$E_{L} = \mathop \sum \limits_{i = 1}^{{N_{e} }} \frac{{k_{L} }}{2}(l_{i} - l_{0,i} )^{2}\quad (5)$
Surface area incompressibility		$E_{A} = \mathop \sum \limits_{i = 1}^{{N_{t} }} \frac{{k_{A} }}{2}\left( {A_{i} - A_{0,i} } \right)^{2}\quad (6)$
Bending resistance	$E_{b} = \mathop \sum \limits_{i = 1}^{N} \frac{{k_{b} }}{2}\tan^{2} \left( {\frac{{\theta_{i} - \theta_{0,i} }}{2}} \right)\quad (7)$	$E_{B} = \mathop \sum \limits_{i = 1}^{{N_{e} }} \frac{{k_{B} }}{2}\tan^{2} \left( {\frac{{\theta_{i} - \theta_{0,i} }}{2}} \right)\quad (8)$
Volumetric incompressibility	$E_{a} = \frac{{k_{a} }}{2}\left( {\frac{{A - A_{ref} }}{{A_{ref} }}} \right)^{2} \quad (9)$	$E_{V} = \frac{{k_{V} }}{2}\left( {\frac{{V - V_{ref} }}{{V_{ref} }}} \right)^{2}\quad (10)$

The force–deformation behaviour of spectrin follows a saw-tooth pattern at the molecular level due to the sudden folding and unfolding of the molecular domains [44]. However, when cytoskeletal behaviour is observed on a larger scale, the fluctuations can be simplified to a linearly increasing trend for force versus deformation. This is equivalent to a harmonic energy potential and is suitable due to the coarse-graining of the membrane. The harmonic type of potential also reflects the bilayer’s resistance to changes in surface area. Thus, in 2D both mechanisms are modelled with a combined linear interaction between adjacent particles as shown in Fig. 1a. The total energy stored via these means, *E*_*l*_, was calculated with Eq. 4, where *i* is the interaction number, *k*_*l*_ is the combined linear stiffness coefficient, *l*_*i*_ is the actual distance between the adjacent particles, and *l*_0,*i*_ is the relaxed distance between adjacent particles. The distance between particles in the initial circular configuration was used for the relaxed lengths.

In the 3D model, stretch resistance and surface area incompressibility are separated. The stretch component is applied between particles joined by edges of the mesh, while the surface area constraint is applied to each individual triangle. Some previous models have also applied an additional energy term to restrict global surface area of the cell [35, 42, 43, 45, 46], however the split between the local and global components was arbitrarily set [47] and stiffness coefficients were selected to simply ensure negligible variation in surface area [43]. Thus, the global surface area constraint is omitted in the present study, consistent with Wu et al. [48]. The energy stored via stretch (*E*_*L*_) was calculated using Eq. 5 in the same way as 2D with a linear stiffness coefficient *k*_*L*_, while the energy stored due to areal incompressibility (*E*_*A*_) is given by Eq. 6. Here $k_{A}$ is the stiffness coefficient for areal incompressibility, *A*_*i*_ is the area of the *i*th triangle and *A*_0,*i*_ is the relaxed area of this triangle. The relaxed area for each triangle and linear interaction lengths were equal to those in the initial sphere. The area of each triangular surface was calculated using the method set out in Polwaththe-Gallage et al. [49].

The bilayer is responsible for resisting bending of the membrane. Therefore, bending potentials were introduced between adjoining particles (2D) and triangular surfaces (3D). For the 2D model, the energy stored in bending (*E*_*b*_) is given by Eq. 7, where *k*_*b*_ is the bending stiffness coefficient, *θ*_*i*_ is the actual angle away from the horizontal (see Fig. 1a) and *θ*_0,*i*_ is this angle relaxed. For the 3D model, energy stored in bending (*E*_*B*_) is given by Eq. 8, where *k*_*B*_ is the bending stiffness coefficient, *θ*_*i*_ is the angle formed between vectors normal to the adjoining triangular surfaces (see Fig. 1b) and *θ*_0,*i*_ is again the relaxed angle. The relaxed angle was set to zero in both models [35, 42].

A volume requirement was imposed on the RBC to model the internal fluid’s incompressibility. In 2D, this manifests as a requirement on the cross-sectional area. The desired cross-sectional area of the RBC, *A*_*ref*_, was calculated from *A*_*ref*_ = *R*_*A*_ *A*_*circle*_ where *R*_*A*_ is the swelling ratio for 2D set to 0.48 for physiological conditions [36] and *A*_*circle*_ is the area of the initial circle. A penalty function enforces this cross-sectional area by storing energy (*E*_*a*_) when actual area (*A*) deviates from the desired area in Eq. 9. Here, *k*_*a*_ is the penalty stiffness coefficient. It represents a “soft” restraint given that the size of the cell can still vary, however the extent is limited if the strength of the penalty is large, thus modelling the incompressibility of the internal fluid. Similarly in 3D, desired volume of the RBC was calculated from *V*_*ref*_ = *R*_*V*_ *V*_*sphere*_, where *R*_*V*_ is the swelling ratio for 3D set to 0.6 for physiological conditions [45] and *V*_*sphere*_ is the volume of the initial sphere. The penalty function for volume (Eq. 10) causes energy to be stored when actual volume of the RBC (*V*) differs from the desired, using a penalty stiffness coefficient, *k*_*V*_. The actual volume of the cell was calculated using the method set out in Polwaththe-Gallage et al. [49].

Finally, if the number of particles used to represent the membrane is changed but stiffness coefficients remain steady, total energy stored in the membrane interactions will change. This will modify how the cell responds to force. In order to overcome this, stiffness coefficients can be normalised against particle number. Therefore for comparison purposes, base coefficients of the form *k*_*base*_ = *k*/*N* are introduced for stiffness coefficients associated with spectrin’s stretch resistance, surface area incompressibility and bending. It should be noted that the stiffness coefficients for maintaining the volume of the cell do not require this treatment as these are applied globally rather than locally. This is regarded as an improved normalisation method as it can be applied in the same manner to each mechanism unlike some previous studies which have only normalised some aspects (e.g. Tsubota et al. [38]).

### Adherence to substrate and indentation

In order to validate the model, the experimental conditions relating to substrate adherence and indentation were replicated (refer to “Experimental method and results” section below). Both AFM and confocal imaging showed that the RBCs formed dome-shapes when adhered. To incorporate this into the model, a constraint was introduced for specific membrane particles to be in contact with the substrate. In 2D, a section of the membrane corresponding to 8.5 µm in length was set to the substrate’s height, while in 3D, 50% of the particles were set to the substrate’s height. Equation 1 was then re-applied to minimise energy and thus predict adhered RBC shape. This treatment of adhesion as a constraint rather than an additional energy term saves computational cost. However, if the model was to be used to specifically study adhesion or detachment, the attraction potential between the membrane and substrate should be quantified with an additional energy term.

**Fig. 2:**
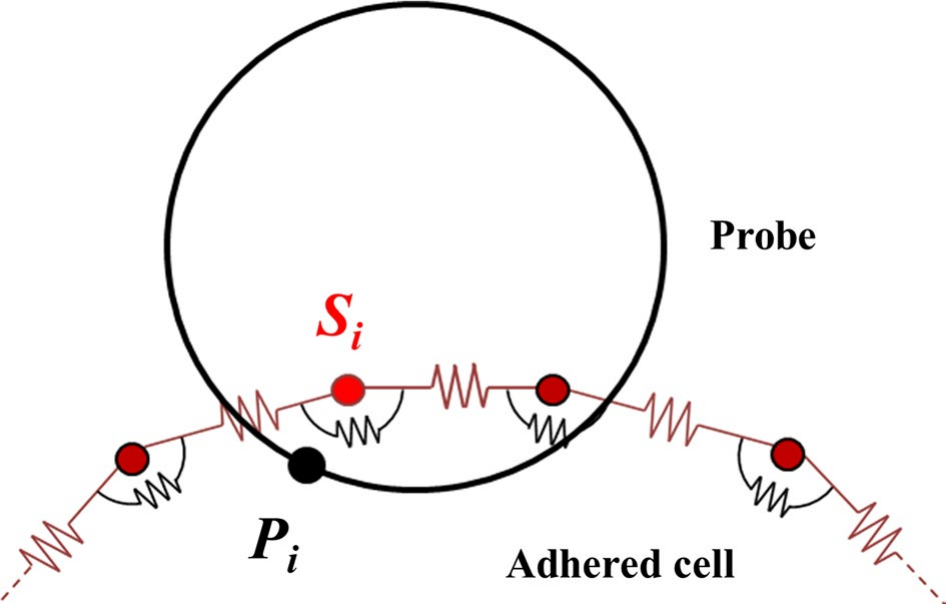
Schematic showing the *i*th membrane particle which has penetrated and the closest point on the probe’s surface

The 5 µm spherical probe was represented as a rigid body (incapable of deforming), given that RBCs are significantly softer. Contact between the probe and cell was modelled with a penalty function,

11 $$E_{con} = \mathop \sum \limits_{i = 1}^{{N_{p} }} \frac{{k_{con} }}{2}\left( {s_{i} - P_{i} } \right)^{2}$$

which stored energy, *E*_*con*_, when cell membrane particles penetrated the probe surface. Here *N*_*p*_ is the number of membrane particles which have penetrated, *k*_*con*_ is the penalty stiffness coefficient for contact, *s*_*i*_ is the position of membrane particle *i* which has penetrated, and *P*_*i*_ is the closest point on the probe’s surface to *s*_*i*_. This is illustrated for clarity in Fig. 2. The penalty stiffness coefficient for contact is a numerical parameter implemented to ensure negligible cross-over of the probe and cell membrane. When it is sufficiently large to enforce the contact, it becomes independent of steady-state RBC shape and measured force. However, when too large, it can cause numerical oscillation requiring particle movements to be slowed and causing an increase in computation time. Thus, a sensitivity study was performed to select the contact stiffness coefficient. This found $k_{con} = 10^{20} \times k_{b} \;\text{m}^{ - 1}$ and $k_{con} = 10 \times k_{b} \;\text{m}^{ - 1}$ to be suitable for 2D and 3D respectively.

To simulate indentation, the probe was centred above the adhered cell and moved down a specified distance (zero position was defined as where contact initiated). Equation 1 was re-applied with the additional energy term, *E*_*con*_, to minimise total energy. This treats the indentation as a “quasi-static” problem, justified by the indentation speed being slow enough that the system remains in internal equilibrium. Total contact force between the cell and probe was measured when the simulation had reached steady-state. By Newton’s Third Law, the measured contact force is equivalent in magnitude to the force applied downwards by the probe to cause the deformation. Indentation was simulated at a series of depths between 0 and 200 nm corresponding to the range tested in the experiments. Contact force and indentation depth were plotted against each other and compared against the experimental reference curve.

### Sensitivity study for particle number

In order to select the number of particles for discretisation of the membrane, sensitivity simulations were completed for both the resting and adhered cases as a function of particle number. An adaptive discretisation technique was utilised to speed up the convergence process, which involved converging the model for a small particle number and then including additional particles at the midpoint of each linear interaction. In the 2D model, this caused the number of particles to be doubled, while in the 3D model this almost quadrupled the particle number each time. The model was then converged again from the pre-existing solution which was significantly faster than starting from the circular and spherical shapes each time the particle number was refined. It should be noted that the parameters referenced from the initialised geometry (such as *l*_*o*,*i*_ and *A*_*o*,*i*_) were obtained as if the model started with the corresponding particle number so no compounding error was introduced from this adaptive discretisation technique.

From the 2D sensitivity study, 400 particles was chosen as the point where dimensions and energy sufficiently stabilised—doubling the number of particles from here resulted in less than a 1% change to the critical dimensions. An equivalent sensitivity study was conducted for the 3D model. This found *N* = 1922 to be most suitable for the 3D indentation simulations.

## Experimental method and results

### RBC samples

Units of leukodepleted packed RBCs in saline-adenine-glucose-mannitol (SAGM) solution were obtained through the Australian Red Cross Blood Service Processing Centre (Kelvin Grove, Australia). Samples from four different units were used in this study. The units were stored at 4 °C under standard conditions.

### Atomic force microscopy

#### AFM probes

In order to observe the behaviour of the membrane within physiological limits, spherical indenters were used [14, 15, 50]. Their smoothness reduces the potential for penetration, rupture and non-physiological localised strains [50]. It has been reported that spherical probe diameters have limited to no influence on the measured Young’s modulus [51, 52] whereas a hundred-fold variation can be found in literature for indentation using sharp tips [9, 10, 12, 13].

Spherical indenters were assembled using Hydra2R-100NG tipless cantilevers (AppNano, Mountain View, USA) and melamine beads of 5 µm diameter (Sigma-Aldrich, Sydney, Australia) and a standard deviation of 0.15 µm. A bead was attached to the tip of the cantilever using a two-part epoxy glue. Placement of the bead was controlled using the AFM piezo electric manipulator.

#### Adhesion to substrate

Due to the scanning step, used to reliably align the indentation contact point with its height, AFM indentation was performed after cells were immobilised on the substrate. Cells were incubated at room temperature in phosphate buffered saline (PBS) to allow them to sink and then adhere to poly-d-lysine coated Petri dishes (TPP, Trasadingen, Switzerland). Poly-d-lysine creates positive charges over the substrate that interact with the negative charges found over the RBC membrane. To minimise tension in the uppermost part of the cell membrane against the need for sufficient adhesion during scanning, a concentration of 100 µg/mL of poly-d-lysine and incubation time of 10 min were chosen.

Glutaraldehyde was used to stabilise the membrane and prevent spreading on the surface while indentation was performed: following incubation with poly-d-lysine, the cells were incubated for 30 s in 1% glutaraldehyde in cacodylate buffer 1% (ProSciTech, Kirwan, Australia). The short incubation time as well as the very low concentration of glutaraldehyde were expected to stabilise the membrane surface proteins. PBS was used for AFM analysis.

#### Indentation

A NanoSurf FlexAFM with NanoSurf C3000 software (NanoSurf, Liestal, Switzerland) was used to indent the samples (n = 26 cells). The RBC surface was first scanned to identify the cell’s shape profile and then indented following a grid pattern to measure the deformation response. Maximum indentation force was set between 0.5 and 2.5 nN and resulted in deformations of less than 200 nm. The deformation depth was kept to less than 10% of cell height, which averaged 2.1 µm, to minimise the effect of the substrate [52]. To further reduce the substrate’s impact on the measured force–deformation behaviour, only results from indentation performed at the centre of the cells were considered in the analysis. Indentation at the centre also means that inclination and asymmetry of the contact area between the cell and probe are minimised [53]. Indentation speed was set at 1 µm/s, as measurements at slower speeds may experience sample drift [13]. At a higher speed (above 5 µm/s), the dynamic reaction force from the membrane has been found to influence the extracted elasticity values. Force-height curves were extracted for indentation points at the centre of the cells using the SPIP image processing software (3D Vizualisation Studio, Horsholm, Denmark).

#### Experimental data analysis

As stated in the introduction, Hertz-based models have significant limitations in reasoning solid mechanics contact assumptions. However, they have been widely used to analyse RBC force-indentation results [8–13, 15]. As this study aims to develop a numerical model for RBC indentation, the intention with the experimental data analysis was to validate the measurements against previous studies and then to select an empirical equation which describes the force–deformation trend for reference. This could then be used to benchmark the numerical model’s performance.

The force-height curves were analysed using MATLAB (MathWorks, Natick, USA). The effective Young’s modulus was extracted by minimising the root-mean-square (RMS) error between the data and the Hertz equation modified by Dimitriadis et al. [52] for spherical tip shape and which corrects for finite sample thickness,

12 $$\begin{aligned} F &= \frac{16}{9}ER^{0.5} \delta^{1.5} \left[ {1 + 1.133\chi + 1.283\chi^{2} + 0.769\chi^{3} + 0.0975\chi^{4} } \right] \hfill \\ & \quad \quad {\text{where}}\quad \chi = \frac{{\left( {R\delta } \right)^{0.5} }}{h}. \hfill \\ \end{aligned}$$

Here *F* is the applied force, *E* is the effective Young’s modulus, *δ* is the indentation depth, *R* is the indenter radius and *h* is the cell height.

### Confocal imaging

Confocal imaging was used to quantify the diameter of adhered cells for replication in the numerical modelling. Briefly, the cells were first incubated for 15 min with DiI (Thermo Fisher Scientific, Scoresby, Australia) and then adhered to a poly-d-lysine coated substrate following the AFM sample preparation protocol, without glutaraldehyde. Imaging was done using a Leica TCS SP5 confocal microscope at exactly 10 min after the beginning of incubation (Leica, North Ryde, Australia). A side view of a typical stack can be seen in Fig. 4. Nine cells were imaged using confocal microscopy following the experimental adhesion protocol. The average substrate contact area was found to be 54.7 µm^2^.

### Experimental results and discussion

**Fig. 3:**
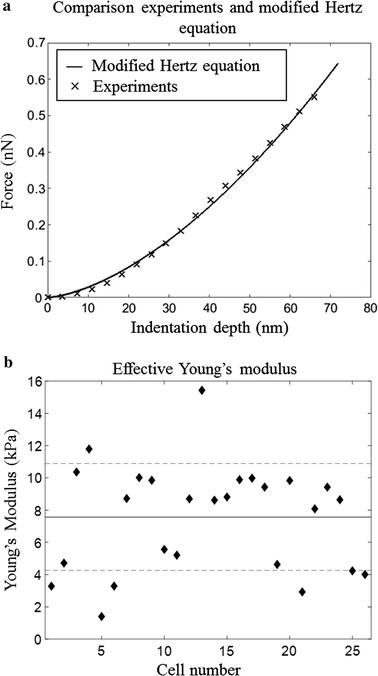
**a** Comparison between experimental data and the modified Hertz equation for a typical sample where E = 9.83 kPa, **b** effective Young’s modulus for each cell; the mean is 7.57 kPa (solid line) with a standard deviation of 3.25 kPa (dotted lines)

The experimental data was observed to closely fit the force–deformation trend predicted by the modified Hertz equation (Fig. 3a). Effective Young’s modulus for each cell is shown in Fig. 3b. The average was found to be 7.57 kPa with a standard deviation of 3.25 kPa (experimental data is provided in Additional file 1). This aligns with previous studies investigating the effective Young’s modulus of the RBC membrane which have reported values between 0.1–0.2 kPa [15] and 98 ± 17 kPa [11]. The wide range is attributed to the differences in sample preparation and indentation protocols, as well as analysis methods (supplementary information in Ciasca et al. [13] contains a comprehensive summary of these differences).

Adding glutaraldehyde is known to have an effect on the membrane function and consequently studies using this method have tended to report a higher Young’s modulus (between 26 ± 3 kPa [8] and 98 ± 17 kPa [11]), compared to those who do not (between 0.1–0.2 kPa [15] and 4.9 ± 0.5 kPa [10]). However, other parameters such as indentation location, probe geometry and indentation speed, to name a few, were also linked to large variation in the calculated Young’s modulus numerical value. This has a significant impact on the interpretation and comparison of quantitative values, with most studies only reporting an average. Regarding indentation location, Ciasca et al. [13] found an effective Young’s modulus significantly higher for the central region compared to near the edges. In fact, for a “typical” cell, Young’s modulus was as high as 9 kPa at the centre, as low as 0.06 kPa near the edge, and 1.87 kPa when averaged over the surface. This study was not the only one reporting these variations [8, 11, 13]. Probe geometric differences also have a substantial impact on measured Young’s modulus, as local strains imposed on the membrane by sharp probes may exceed physiological levels and subsequently trigger a reorganisation of the membrane structure. Different probes also mean that different Hertz equations have been applied in an attempt to take into account the geometry, with some neglecting modifications for finite sample thickness and substrate effects. Thus, while acknowledging complicating factors in the comparison, the result of the present experiment is comparable with existing data. It is also shown that the experimental data follows the trend predicted by the modified Hertz equation. This gives strong support for the development of the numerical model, using the present experimental result as a reference, which extends into the explanation of mechanical behaviour of the RBC membrane.

## Model validation

The mean value for effective Young’s modulus from the experiments was used within the modified Hertz equation to validate the force–deformation behaviour of the model. This is because of the variability in the value measured for different cells but each closely followed the trend of the modified Hertz equation.

An inverse method was applied to extract the stiffness coefficients that best predict RBC shape (resting and adhered) and the force–deformation behaviour for indentation. Initial values were assumed for each stiffness coefficient which were then iteratively converged until reasonable agreement was reached between the model and experimental observations for resting shape, adhered shape and indentation behaviour. In 2D, the optimised stiffness coefficients were $k_{l} = 1.2 \times 10^{2} \;\text{N}/\text{m}$, $k_{b} = 1.6 \times 10^{ - 14}\;\text{J}/\text{rad}$ and $k_{a} = 2.3 \times 10^{ - 10} \;\text{J}$. Similarly, in 3D, the optimised stiffness coefficients were $k_{L} = 2.1 \times 10^{ - 4} \;\text{N}/\text{m}$, $k_{B} = 5.3 \times 10^{ - 15} \;\text{J}/\text{rad}$, $k_{A} = 1.4 \times 10^{11} \;\text{N}/\text{m}^{2}$, and $k_{V} = 7.0 \times 10^{ - 10} \;\text{J}$.

**Fig. 4:**
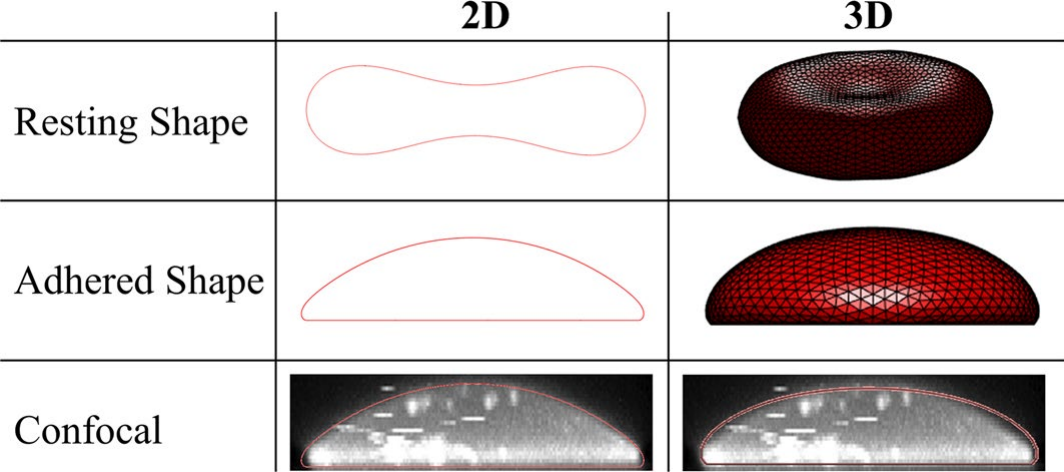
2D and 3D predictions for resting and adhered RBC shape, including comparison to typical confocal image

The predicted resting and adhered cell shapes are shown in Fig. 4. Critical dimensions for the RBCs at rest are each within 10% of those reported by Evans et al. [54], while the adhered RBCs have a diameter and height within 10% of the mean dimensions obtained in the present experiments. Qualitatively, the cross-sectional shape matches well with the confocal results.

**Fig. 5:**
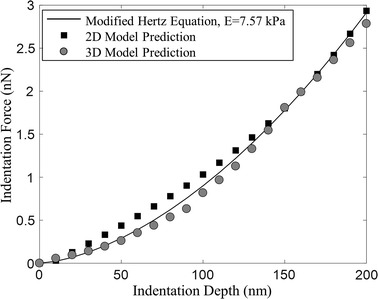
Force-deformation prediction of the 2D and 3D models validating performance

The force–deformation curves predicted by the numerical models using the optimised stiffness coefficients are shown in Fig. 5. These can be compared against the modified Hertz equation with effective Young’s modulus of 7.57 kPa. Overall, good agreement is reached over the investigated range. However for the 2D case there is some discrepancy in the early deformation region where the model predicts a more linear trend and a larger force than in the experimental case. The 3D model is able to better capture the behaviour through the early region.

## Results and discussion

### Mechanisms through indentation

**Fig. 6:**
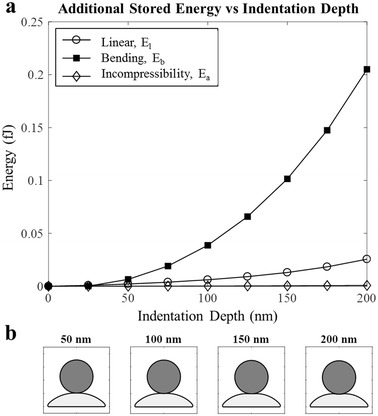
**a** Energy and **b** cell shape through indentation stroke

In order to understand which mechanisms impact on the cell’s behaviour at different indentation depths, cell shape through the indentation stroke is shown in Fig. 6b. For the early stages, it can be seen that the cell’s surface flattens out under the application of the force. Beyond this, a concavity is introduced into the surface by the probe. Consequently, at small indentation depths, indentation force is mainly used to modify curvature of the membrane, impacting energy stored through the bending mechanism. With further indentation, the probe causes the membrane to curve beneath and at the same time, the cytoplasm needs to redistribute to avoid being compressed. Consequently the cytoplasm applies pressure on the membrane, which in turn develops tension. Thus, in addition to a change in the bending energy, there is also an increase in energy stored through membrane tension. This trend is evident in both the 2D and 3D results, however it is most clear in Fig. 6a which shows the additional energy absorbed in each mechanism for the 2D simulation. It would be expected that for deeper indentations, tension on the membrane would become dominant over bending stiffness, as the cytoskeleton is stretched.

As bending is most influential, especially at small deformations, the overestimation for the force in the 2D model at these small deformations is most likely caused by the underlying assumption for the quantification of bending energy. Currently, energy developed in bending interactions is directly proportional to the tangent squared relationship of the angle. This appears to capture the behaviour well in the 3D model, but introduces a small error in 2D. In order to better capture the trend and magnitude, a variable bending stiffness coefficient may be considered for the 2D model to compensate for the 2D simplification. Variable stiffness coefficients have been implemented in previous 3D models for the linear stretch mechanism [45, 47], but have not been attempted for bending before. This may be a future research focus to improve the 2D model for greater accuracy where a lower computational cost is important for the application.

### Effect of modifying stiffness coefficients

**Fig. 7:**
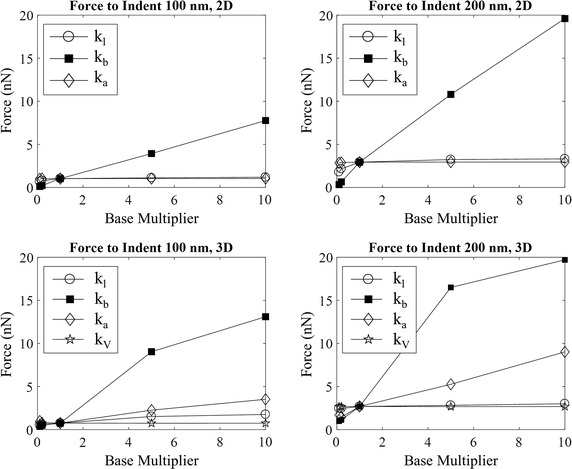
Parametric study measuring indentation force when varying stiffness coefficients in both 2D and 3D

In order to explore the influence of the individual mechanisms involved in RBC deformability, a parametric study was conducted for the stiffness coefficients. This was performed by varying each stiffness coefficient in isolation to between a tenth and ten times the baseline value established during the validation. Indentation was then simulated to nominal depths of 100 and 200 nm. The results are shown in Fig. 7.

It can be seen for the 2D cases, varying the membrane’s bending stiffness causes a significant change in the deformability of the RBC. In fact, a roughly linear relationship is present between the force required to indent the cell and the bending stiffness coefficient. In contrast, the effect of the linear stiffness coefficient is minimal, particularly as it is increased from the baseline multiplier of 1. This means that further increases have very little effect on the RBC’s deformability. The stiffness coefficient for incompressibility of the internal fluid has a negligible impact.

For the 3D cases, changing the membrane’s bending stiffness still has the most substantial impact on deformability, however it starts to plateau unlike the 2D model. The linear and areal stiffness coefficients have a small influence when indenting to 100 nm, with the influence of the areal stiffness coefficient becoming more important at the deeper indentation depth. This aligns with the finding in the previous section that tension in the membrane becomes more significant with larger indentation depths. The negligible impact of the volumetric incompressibility coefficient agrees with the 2D result.

These results demonstrate that overall deformability of the RBC is most sensitive to changes in bending stiffness. As bending resistance is provided by the membrane’s outer lipid bilayer and its embedded proteins, this suggests structural changes within this part of the membrane play the most critical role in the loss of deformability observed in deteriorating RBCs.

### Comparison to stiffness coefficients of previous studies

A literature review was conducted on previous studies employing the CGPM to model RBC physical behaviour. All models identified and presented in Table 2 only considered flows, with the exception of Shi et al. [42] which also considered stretching. Areal incompressibility stiffness coefficients are not presented as most models used both local and global constraints for which an equivalent combined value cannot be computed. Significant variation can be seen in the stiffness coefficients, suggesting there is a range of values which may be suitable for simulating RBC behaviour. The values selected for this study lie within the range used previously. Values presented in Table 2 are normalised against the number of particles used to represent the membrane, for fair comparison.

**Table 2:** Literature review of parameters for RBC simulations using coarse-grained particle method applied in flows, normalised against particle number

2D paper	Case	N (particles)	r (µm)	$k_{l,base}$ (N/m/particle)	$k_{b,base}$ (J/rad/particle)	$k_{a}$ (J)
Tsubota et al. [38]	–	76	3.0	1.1E+04	6.6E−12	1.0E−05
Wang et al. [40]	Min	76	2.8	2.5E−02	1.3E−15	1.0E−09
	Max			2.5E−01	1.3E−14	1.0E−08
Pan et al. [56]	Min	76	2.8	2.5E−03	1.3E−16	1.0E−10
	Max			7.4E−01	3.9E−14	3.0E−08
Shi et al. [22]	–	76	2.8	1.2E+04	6.6E−12	1.0E−05
Wang et al. [57]	Min	76	2.8	2.5E−04	1.3E−17	1.0E−11
	Max			1.2E−02	6.6E−16	5.0E−10
Tsubota et al. [41]	Min	48	3.0	2.7E+01	4.2E−13	2.0E−07
	Max			2.7E+03	4.2E−11	2.0E−05
Shi et al. [36]	–	76	2.8	1.2E+04	6.6E−12	1.0E−05
Polwaththe-Gallage et al. [33, 58]	–	88	2.8	1.4E+04	5.7E−12	1.0E−05
Polwaththe-Gallage et al. [34]	–	88	2.8	8.5E−01	3.4E−14	3.0E−08
Wang et al. [55]	Min	76	2.8	7.4E−02	3.9E−15	3.0E−09
	Max			7.4E−01	3.9E−14	3.0E−08
Present study	–	400	3.0	7.8E−04	9.8E−20	2.3E−10

3D paper	Case	N (particles)	r (µm)	$k_{L,base}$ (N/m/particle)	$k_{B,base}$ (J/rad/particle)	$k_{V}$ (J)
Tsubota et al. [43]	–	2304	3.27	2.6E+04	5.6E−22	1.8E−16
Nakamura et al. [45]	–	Not stated	3.25	Variable stiffness	Not calculable	4.3E−15
Shi et al. [42]	Min	770	3.28	7.1E−09	2.9E−21	4.7E−15
	Max			9.7E−09	4.1E−21	4.7E−15
Wu et al. [48]	1	440	2.8	2.7E−08	3.2E−21	Not stated
	2	1058		1.1E−08	1.3E−21	Not stated
Nakamura et al. [47]		2648	3.27	Variable stiffness	3.8E−06	4.4E−15
Polwaththe-Gallage et al. [35, 46]	–	954	3.1	1.6E−08	2.0E−20	3.7E−15
Present study	–	1922	3.3	1.1E−07	2.8E−18	7.0E−10

**Fig. 8:**
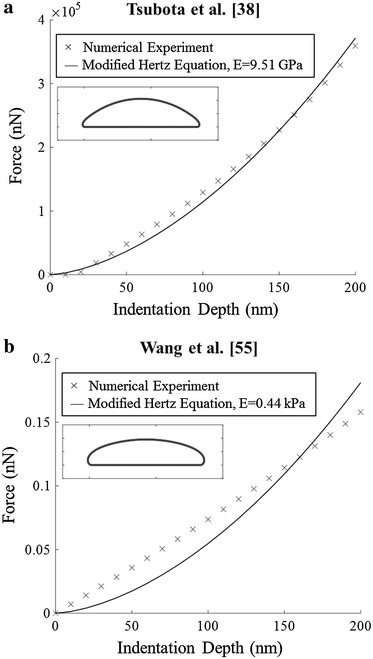
Force deformation curves obtained using parameters from **a** Tsubota et al. [38] and **b** Wang et al. [55]. Insert show the adhered cell geometry

In order to test whether previous values would also be appropriate for indentation, a mid-range (Wang et al. [40]), large (Tsubota et al. [38]), and small (Wang et al. [55]) set of stiffness coefficients from Table 2 were tested for indentation (Fig. 8). For the mid-range and small set, the adhered cell shapes were much flatter than the confocal results and there was little agreement between the force–deformation trend of the model and the modified Hertz equation (Fig. 8b). The large set showed reasonable agreement for the adhered shape and force–deformation trend (Fig. 8a), however the best-fit Young’s modulus was 9.51 GPa, significantly larger than literature reports [13].

These findings demonstrate the stiffness coefficients extracted in this study are more suitable for modelling indentation of RBCs. It also suggests indentation simulations are more sensitive to stiffness coefficient selection. This is because flow studies use qualitative comparison of RBC shapes in general flow conditions, whereas the indentation model uses both qualitative RBC shapes and quantitative force–deformation response. The quantitative aspect means the system’s response is more sensitive to changes in the stiffness coefficients—if only qualitative comparison of shape is considered, modification of the stiffness coefficients which have little impact on cell shape cannot be detected. This positions indentation as a preferred model for investigating the impact on deformability caused by changes within the RBC membrane as they will be measured far more readily.

## Conclusions

This study developed an effective protocol for measuring the mechanical properties of RBCs, utilising AFM with a spherical probe in liquid. The force–deformation behaviour was shown to follow the modified Hertz equation for finite thickness samples and provided a measurement of the Young’s modulus for the model of 7.57 ± 3.25 kPa, consistent with literature reports. A numerical model based on the coarse-grained particle method (CGPM) was developed for simulating RBC deformation behaviour during indentation in both 2D and 3D for the first time, and achieved good agreement with the experimental observations. The models were applied to investigate the mechanisms which absorbed energy through the indentation stroke, and the impact of varying stiffness coefficients on the measured deformability. This found the membrane’s bending stiffness was most influential in controlling RBC physical behaviour for indentations of up to 200 nm. As the bilayer provides bending resistance, this infers that structural changes within the bilayer are responsible for the deformability changes experienced by deteriorating RBCs. This indentation model forms a foundation for future investigations into structural changes within the membrane and how these impact on cellular deformability, given that differences in stiffness have been shown experimentally between healthy and deteriorating RBCs. This CGPM model has significantly more versatility in simulating RBC indentation as it considers the membrane’s resistance to bending, stretch, areal changes and volume changes, as well as affording control over the shape and dimensions of the probe, adhesion of the membrane to the substrate, and the direction and position of the applied indentation force. The numerical model presented here established a foundation for future investigations into changes within the membrane that cause differences in stiffness between healthy and deteriorating RBCs, which have already been measured experimentally with AFM.

## Additional file

**Additional file 1.** Experimental data.

## Abbreviations

RBCs: red blood cells
AFM: atomic force microscopy
CGMD: coarse-grained molecular dynamics
CGPM: coarse-grained particle method
FEM: finite element method
SAGM: saline-adenine-glucose-mannitol
