# Supplementary material for: Investigation of red blood cell mechanical properties using AFM indentation and coarse-grained particle method
Source: Biomed Eng Online. 2017 Dec 19;16:140. doi: 10.1186/s12938-017-0429-5 (PMC5738115; doi:10.1186/s12938-017-0429-5)
Supplement: Supplementary file 1 — Additional file 1. Experimental data. [file 12938_2017_429_MOESM1_ESM.pdf]

Barns, et al. Supplementary information – experimental data

Number of indentations (grid)	Number of centre points selected	Young's Modulus (Mean)	Young's Modulus (SD)	Sample
**64 (8 x 8)**	16	2927.39	699.12	1
**64 (8 x 8)**	18	3283.69	1238.95	1
**64 (8 x 8)**	19	3288.75	1311.71	1
**64 (8 x 8)**	20	4010.70	1374.16	1
**64 (8 x 8)**	17	4237.43	1214.20	1
**64 (8 x 8)**	14	8616.91	582.28	1
**64 (8 x 8)**	14	8718.70	659.43	1
**144 (12 x 12)**	16	8814.73	600.02	1
**64 (8 x 8)**	14	9426.47	643.44	1
**64 (8 x 8)**	14	9428.29	730.66	1
**64 (8 x 8)**	14	9851.85	465.83	1
**64 (8 x 8)**	15	9982.72	921.01	1
**64 (8 x 8)**	13	10361.21	1285.56	1
**144 (12 x 12)**	26	8700.29	1132.03	2
**144 (12 x 12)**	26	9833.08	852.67	2
**144 (12 x 12)**	31	4716.67	1366.96	2
**64 (8 x 8)**	24	5206.29	1046.41	3
**64 (8 x 8)**	15	8644.05	457.31	3
**64 (8 x 8)**	8	4632.97	270.90	3
**64 (8 x 8)**	11	11788.77	1893.27	3
**64 (8 x 8)**	12	1402.82	63.03	4
**64 (8 x 8)**	8	5565.44	249.00	4
**64 (8 x 8)**	24	8081.06	425.88	4
**64 (8 x 8)**	12	15431.17	1804.12	4
**144 (12 x 12)**	17	9887.31	1527.53	4
**144 (12 x 12)**	25	10015.77	255.49	4
